# Understanding the Factors Affecting Sustainable Energy Action Plan: A Case Study From the Covenant of Mayors Signatory Municipality in the Aegean Region of Turkey

**DOI:** 10.3389/fpsyg.2021.635980

**Published:** 2021-05-25

**Authors:** Mehmet Efe Biresselioglu, Muhittin Hakan Demir

**Affiliations:** ^1^Sustainable Energy Division, İzmir University of Economics, İzmir, Turkey; ^2^Department of Logistics Management, İzmir University of Economics, İzmir, Turkey

**Keywords:** Covenant of Mayors, municipality, sustainable energy action plan, energy transition, İzmir, Turkey

## Abstract

This study presents the case of a Metropolitan Municipality in the Aegean Region of Turkey, which undertook a series of initiatives to conduct projects on environmental protection and sustainability. This case study was conducted as two separate studies as a part of Horizon 2020-funded ECHOES project under Work Package 6, aiming to gain insight into the collective magnitudes of energy-related choices and behavior. The starting point of the process is marked, in 2015, by the municipality becoming a party to the Covenant of Mayors movement, joining around 8,000 signatories from over 50 countries. In line with European Union’s (EU’s) climate targets and associated energy-related policies, signatories of the Covenant of Mayors aim to decrease carbon emissions by 20% by 2020 and by 40% by 2030. In order to enhance the design and operationalization of policies for achieving these targets, each partner in the Covenant of Mayors is required to develop a Sustainable Energy Action Plan (SEAP). The SEAP is to be prepared within 2 years of becoming a party to the Covenant of Mayors and involves action plans and projects in order to operationalize the strategies for achieving the associated targets. To this end, this study analyzes the Metropolitan Municipality’s SEAP and its components, which include zero-emission public transportation project, transformation of existing buildings to a more energy-efficient standard and related energy audit studies, a project for decreasing waiting periods in traffic *via* a Smart Traffic System, a pedestrianization project, and a project for increasing the use of geothermal energy for district heating. This study set out to identify the internal and external factors, as well as bottom-up and top-down mechanisms involved in various phases of the preparation and implementation of the SEAP. The research method was expert interviews, incorporating viewpoints and perceptions of stakeholders from different levels of the municipality. Among the key results are understanding the roles of enthusiasts and frontrunners in such initiative and the importance of top-management and central government support.

## Introduction

In the last decade, the significance and impacts of environmental concerns have become globally recognized. The adverse effects of global warming and climate change are increasingly visible as a result of globalization, growing industrial activity, and urbanization. Adding to these, the rapidly growing population poses risks to the availability of food, water, and energy resources including fossil fuels. These issues have serious consequences for the environment and humans (UN, 2018). One of the foremost responses to these challenges and risks is the adoption of sustainable development policies. These policies call for central and local governments to become parties to agreements that involve targets and commitments regarding environmental sustainability issues, such as considerably reduced carbon emissions and participation in environmental protection efforts. The Kyoto Protocol of 2005 depends on countries’ commitments to decrease greenhouse gas emissions. Similarly, the countries that are the signatories of Paris Agreement, in effect as of 2016, committed to efforts to mitigate the effects of climate change and implement strategies that will help developing countries, as well as the signatory countries themselves, to adapt to the effects of climate change.

The signatories of the Paris agreement are the national governments themselves. However, climate change and adaptation efforts are not solely the domain of central governments; local governments, communities, non-governmental organizations (NGOs), and industry clearly have significant roles and responsibilities. Owing to their local power, and the development of leaner and more dynamic structures, local governments have implemented many initiatives regarding environmental issues and sustainability. One striking example is the Covenant of Mayors movement. The Covenant was established in 2008, and the local authorities in the movement share the ambitious goal of reaching, and even exceeding the climate and energy targets of the European Union (EU) (Covenant of Mayors Website, 2020). The Covenant of Mayors aims to harness local governments’ effectiveness, rapid decision-making structures, and shorter lead times in implementing decisions to address climate change. In this respect, the Covenant of Mayors enhances the uptake of bottom-up approaches by local governments in decision-making processes and implementations (Energycities, 2018).

As of 2020, the Covenant of Mayors movement has around 8,000 signatories from over 50 countries. In line with EU’s climate targets and associated energy-related policies, the signatories aim to decrease their carbon emissions by 20% by 2020 and 40% by 2030. In order to enhance the design and operationalization of policies for achieving these targets, the Covenant of Mayors involves each partner developing a Sustainable Energy Action Plan (SEAP). The SEAP needs to be prepared within 2 years of becoming a party to the Covenant of Mayors, and it involves action plans and projects to operationalize the strategies for achieving the associated targets (Covenant of Mayors Website, 2020).

The Metropolitan Municipality of Izmir, the major Aegean Region city, signed the Covenant of Mayors in 2015. The municipality is among 16 signatories from Turkey, three of which are Metropolitan Municipalities (Covenant of Mayors Website, 2020). This case study focuses on the process of the SEAP for the Metropolitan Municipality (Izmir SEAP, 2016). The city was selected because the municipality demonstrated keen interest on this initiative, as well as similar initiatives in the past. The Metropolitan Municipality is also a member of World Health Organization’s European Healthy Cities Network, which aims at emphasizing the role of health within local governments’ strategies and operations, considering the social, economic, and political aspects of health-related issues. The municipality also established a separate department, the Branch Office of Healthy Cities and Clean Energy, responsible for coordinating the strategies and operations for environmental initiatives and mitigating climate change.

The effects of climate change and environmental issues are critical for Turkey, as for any country (Biresselioglu, 2012). However, considering the high share of fossil fuels in Turkey’s energy mix and its great dependence on energy imports, initiatives to tackle climate change have multidimensional significance (Biresselioglu et al., 2017). The share of fossil fuels in Turkey’s energy consumption is 85%, with a 74% dependence on energy imports (Republic of Turkey Ministry of Foreign Affairs, 2019), representing significant risks for energy security. Regarding the energy diversification and sustainability pillars of energy security, increasing the use of renewable energy resources is important in Turkey’s energy strategy agenda (Republic of Turkey Ministry of Foreign Affairs, 2019). The share of renewable sources in Turkey’s energy production is 49%, of which hydro contributes 17.9%; geothermal, 14.8%; biomass, 10.1%; and wind and solar, 3.1% each [International Energy Agency (IEA), 2017].

This perspective on climate change, reduction of carbon emissions, related initiatives, and energy security concerns highlights the role of the concept of energy self-sufficiency and associated initiatives in general, and in particular, for local communities. Such initiatives are also supported by developments in smart and green energy technologies (Biresselioglu et al.,2018a,b).

Municipalities have the responsibility for the planning and operation of public transportation, which is a critical component of overall transportation, especially with increasing population and population density. The transportation sector is responsible for around 25% of the greenhouse gas emissions in Europe, making it a major source of air pollution in cities. Of the various components of transportation, road transport alone is responsible for more than 70% of all Europe’s transport-related greenhouse gas emissions [European Commission (EC), 2019].

To this end, this study analyzes the SEAP and its phases through data from in-depth interviews with expert participants in the SEAP process. In doing so, it identifies the drivers, motivators, and barriers affecting the process. The drivers in various phases of the preparation and implementation of the SEAP include the following: internal and external factors, bottom-up and top-down mechanisms, lower-level dynamics affecting energy choices, and energy-related behavior of the municipalities. The expert interviews reveal the viewpoints and perceptions of stakeholders at different levels of the municipality. The deliberate choice of interviewees to include all decision-making levels enhances the analysis of the institutional and governance framework dynamics.

## Literature Review

The more visible environmental impacts of climate change include increased carbon emissions, resulting in declining air quality and greater noise pollution (Adamou et al., 2012; Colmenar-Santos et al., 2014; Kasten and Hacker, 2014; Da Silva and Moura, 2016; Mahmoud et al., 2013). However, the social attitude and response toward climate change have been affected by the generally gradual and difficult-to-perceive nature of its impacts. This leads to the perception of climate change as a phenomenon whose effects are uncertain and remote in time.

Therefore, in order to enhance the awareness and contribution of citizens for mitigating climate change, policies are needed that emphasize its immediate, local, and observable effects. In addition to increasing awareness, it is important to highlight the responsibilities of citizens regarding their energy consumption behaviors and to enhance individual norms (Steg et al., 2005). This can be achieved by implementing interventions to promote energy conservation and by using appropriate design for communications emphasizing citizens’ roles and responsibilities about energy saving, making use of constructs from behavioral psychology and behavioral economics (Santin et al., 2009; Frederiks et al., 2015; Podgornik et al., 2016).

Lacroix and Gifford (2018) analyzed the relationship between psychological barriers and energy conservation behavior and identified a set of prevalent barriers. They also conclude that different cultural norms and worldviews influence individuals’ perceptions of psychological barriers. In the case of Turkey, for instance, Dursun et al. (2019) conduct a survey with young consumers to investigate the role of environmental awareness in overcoming psychological barriers against energy conservation behaviors. They verify the effectiveness of objective environmental information in this aim.

In the context of behavioral economics, Sütterlin et al. (2011) aim at establishing a segmentation of consumers with respect to energy behaviors, and they propose tailoring efficient policies for specific consumer segments. Good (2019) investigates the potential of behavioral economic theory to provide insights to energy demand response, and he concludes that acknowledging social factors can enhance the management of demand response. Andor and Fels (2018) analyze four categories of non-price interventions aimed at households (social comparison, commitment devices, goal setting, and labeling), concluding that, to varying degrees, all can be effective in developing energy conservation behaviors.

In view of their specific advantages, local authorities emerge as actors and key stakeholders in this process. They are able to actively participate in formulating and implementing strategies toward increasing citizens’ awareness and contributions (Biresselioglu et al., 2020). Sustainable urban development relies heavily on initiatives and actions at the local community level (Comodi et al., 2012; van Doren et al., 2016; Tingey and Webb, 2020), and local governments are becoming increasingly involved in promoting urban sustainability strategies. Hence, the roles of municipalities are undergoing a transformation in their role from local-scale implementers of central government policy toward formulating and implementing their own policies on the local scale. At this point, it is important that local strategies and regulations developed by municipalities conform with the framework of the central governments (Häkkinen et al., 2016). Energy policy development and implementation at the local level requires close coordination with the central government authorities as well as with local stakeholders (Hoppe et al., 2011). However, even with such coordination, financial and regulatory barriers may pose challenges to local governments (Mey et al., 2016; Bergman and Foxon, 2020). Another serious barrier to local authority policies and initiatives is lack of citizens’ awareness and support (Hong et al., 2015; Koirala et al., 2018; von Wirth et al., 2018).

When climate change is considered in the context of cities, the transportation sector is a major component. The transportation sector accounts for 25% of the greenhouse gas emissions of Europe (European Commission (EC), 2017; ICCT International Council on Clean Transportation Website, 2020). To this end, electric mobility (e-mobility) is one of the most important strategies for mitigation of climate change and the energy security of Europe. Advantages triggered by deployment of e-mobility include the improvement of air quality due to reduced use of fossil fuels for transportation, emergence of new business areas related with e-mobility technologies, and job creation (Haddadian et al., 2015; European Commission (EC), 2018). Thus, e-mobility establishes a contemporary sustainability concept in the field of transportation (Peters et al., 2011; Faria et al., 2014). Moreover, e-mobility is a major pillar of the 2030 target of European Commission regarding the achievement of zero-emission urban transportation for freight, and the 2050 target of zero-emission urban transportation for passengers. The increased use of e-mobility for public transportation is an encouraging development, which not only contributes to these future targets but also demonstrates immediate gains on air quality and awareness and support of citizens (Öko Institute, 2012; Barisa et al., 2016; Kaplan et al., 2016).

Clearly, e-mobility is not all without drawbacks. Electric mobility increases the demand for electricity generation, and thus, carbon emissions (Öko Institute, 2012; Kasten and Hacker, 2014). The recycling of electric vehicle batteries also poses environmental threats (Haddadian et al., 2015).

Owing to its economic, political, and environmental facets, energy emerged as an important item in the policies, strategic plans, and programs of many countries. The operationalization of the policymaking on the central government level requires the transposition of the elements of these policies to the local and individual scales; initiatives of local communities and their relationship with central policies and strategies are thus critical. Sperling et al. (2011) investigate to what extent local government initiatives are in line with government strategies, by focusing on the implementations of municipalities in Denmark. van der Schoora and Scholtens (2015) utilize the case study method to reveal how local-scale collective energy initiatives can promote local self-sufficiency, emphasizing the role of the local network, a common vision, and organizational characteristics. Pablo-Romero et al. (2015) concentrate on the municipalities that have undersigned the Covenant of Mayors and identify factors that affect participation in the Covenant of Mayors: the availability of resources for renewable energy, financial considerations, environmental issues, political perspectives of citizens, and previous experience of implementations. Hoppe et al. (2015) analyze the cases of two successful implementations involving local administrations and energy initiatives from Germany and Netherlands. They highlight the importance of leadership, enhanced communication, trust, and process management. Brummer (2018) investigates positive and negative factors affecting community energy initiatives through a comprehensive analysis of implementations in the United Kingdom, Germany, and the United States. The positive factors are economic issues, awareness and acceptance, environmental concerns, achievement of targets, stakeholder participation, and innovation. The main barriers are identified as organizational problems, legal structure, insufficient institutional support, lack of political support, negative attitudes, and lack of organizational capacity. van Doren et al. (2016) consider barriers to general energy conservation initiatives, categorized in terms of under socio-cultural, market, political, and geographical features.

## Materials and Methods

This research was undertaken as a part of Horizon 2020-funded ECHOES project’s Work Package 6, coordinated by MEB (the lead author) and also contributed to D6.3 and D3.1 of the project ECHOES (2017, 2019), in which MEB was also the lead author.

Both authors in this study contributed to these deliverables and, more specifically, were responsible for the “Sustainable Energy Action Plan (Turkey)” and Zero Emission Public Transportation Project (Turkey)” case studies in D6.3 (ECHOES, 2019).

### Methodology

This study utilizes the case study methodology, chosen for its appropriateness, its capacity to explore real life, and contemporary case or cases over time. It relies on detailed, in-depth data from multiple sources, allowing the detailed report of a case description and case themes. This method allows us to study the unit in focus, in the context of a larger group, making the results more verifiable and representative (Creswell, 1998; Creswell, 2013).

The use of multiple sources of information allows for the uncovering of different aspects and various viewpoints of the focal concepts and topics (Baxter and Jack, 2008). Thus, a case study can provide a comprehensive analysis of the research subject (Stake, 1995; Yin, 2006).

This analysis is achieved through working with participants or stakeholders as sources. These sources are directly involved with the case topic, and their viewpoints allow detailed insight into the context and also facilitate understanding of participants’ actions (Lather, 1992; Robottom and Hart, 1993).

A systematic approach to the case study and the presentation of results will enable the identification of similarities and differences among participants, managerial levels, and different perspectives. Hence, the scope of the case study must be carefully determined during the research design (Stake, 1995; Yin, 2003). The scope involves the identification of the definition of the case, the case framework, timeline, place, and activities involved within the case (Miles and Huberman, 1994; Stake, 1995; Creswell, 2003).

To maximize the possibility of generalizing the case level evidence, and providing adequate input for extrapolation, two methodological criteria are considered: representation and contrasting situations (Eisenhardt, 1989).

The case study is conducted through the implementation of qualitative inquiry, in this case, semi-structured in-depth interviews with the relevant stakeholders. In semi-structured in-depth interviews, open-ended questions provide the flexibility to capture respondents’ perceptions and perspectives (DiCicco-Bloom and Crabtree, 2006; Kallio et al., 2016). In an in-depth interview, questions can be fine-tuned to previous contributions (Hardon et al., 2004; Rubin and Rubin, 2005; Polit and Beck, 2010). The method aims to explore the research topic by comparing different perspectives on similar aspects (Holloway and Wheeler, 2010), *via* a common interview template or guideline (Gill et al., 2008). Kvale (1996) lists the steps of the in-depth interview as follows: thematizing, designing, interviewing, transcribing, analyzing, verifying, and reporting. Interviewees were selected using a combination of purposive sampling and convenience sampling. Initially, representatives from each of the three managerial layers of the municipality were selected through purposive sampling according to the aims of the study. Then, convenience sampling method was utilized for the rest of the sample. This method ensured that at least two interviewees were selected from each managerial level for performing in-depth expert interviews, so that all phases of the SEAP process and all viewpoints are covered (Curtis et al., 2000; Taherdoost, 2016).

The choice of the sample, i.e., the number of interviews to conduct, was determined through the requirements of the selected methodology. The main aim of in-depth expert interviews was to obtain a comprehensive understanding of the SEAP and related processes and to identify categories of drivers, motivators, and barriers based on these data, focusing on the relationships between these variables through the experiences of the interviewees undergoing the SEAP process. Therefore, two main factors were considered in the selection of the sample size. The first was to select a sample size to assure that the whole timeline of the SEAP process was covered by the interviewees. The second was to achieve saturation, that is, collecting sufficient data, so that no new data concerning the SEAP process, its drivers, and the relationships between drivers would be contributed by further interviewees. Owing to the homogeneity of data provided by each managerial level, and the purposive sampling, the emergent findings started repeating after six interviews. At this point, sufficient data were obtained through the expert interviews (Morse, 2000; Mason, 2010; Baker et al., 2012).

**FIGURE 1:**
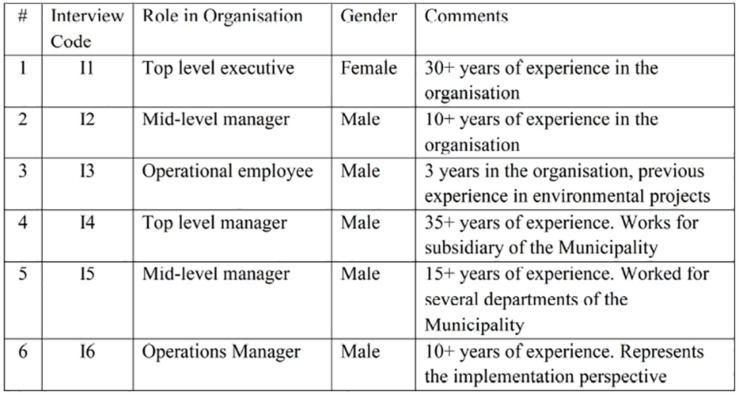
A summary of interview characteristics.

Accordingly, the interview stage involved six different interviewees representing different levels of the municipality. Their profiles are presented in Figure 1. Interviews were conducted in 2017 and 2018.

The interview protocol was prepared in sections covering all phases and all aspects of the SEAP (Hollway and Jefferson, 1997; McCormack, 2004). For this purpose, the interview questions guided interviewees toward a case study narrative, following the process phases in chronological order, focusing on the categories of drivers, motivators, and barriers at each step. The main sections of the interview protocol were as follows: descriptions of the case and the role of each stakeholder, analysis of the current approach and alternatives, planning and development of the roadmap, and implementation and implementation results from stakeholder perspectives. Finally, the interviewees were asked for general comments, suggestions, and recommendations.

The analysis of the transcripts of the in-depth interviews was conducted first using open coding as an initial analysis to identify and extract themes. The second step was the use of axial coding to establish the relationships and interactions between the themes identified *via* open coding. As the final step, selective coding was utilized to refine the findings of open and axial coding to determine the main themes for each phase, representative themes for key internal dynamics, and external factors affecting each phase, along with motivators and barriers (Flick et al., 2004; Shenton, 2004).

Triangulation, using different data sources and collection methods to reinforce the robustness and solidity of the analysis, is also crucial for a successful analysis. To achieve this, interviews were conducted with six interviewees in each organization, including a top-level executive, mid-level managers, and operational/field employees. This both enhances the analysis and contributes to successful triangulation through the collection of data from sources that reflected different aspects of the case, and through the independent analysis of data and results by multiple researchers (Flick, 1992).

## Results

The SEAP of Izmir Metropolitan Municipality includes several components: zero-emission public transportation project, transformation of buildings from the existing standard to a more energy-efficient standard and related energy audit studies, a project for decreasing waiting period in the traffic *via* a Smart Traffic System, pedestrianization project, and a project for increasing the use of geothermal energy for district heating (Izmir SEAP, 2016). Among these, zero-emission public transportation project is of particular importance, involving the increased use of renewable energy, decreased carbon emissions in transportation, and local communities’ energy self-sufficiency implementations. Therefore, the following analysis places special emphasis on this project. The project involves the establishment of a fleet of 400 electric public buses, including the charging and maintenance infrastructure, and also a solar power production system for the required power.

**FIGURE 2:**
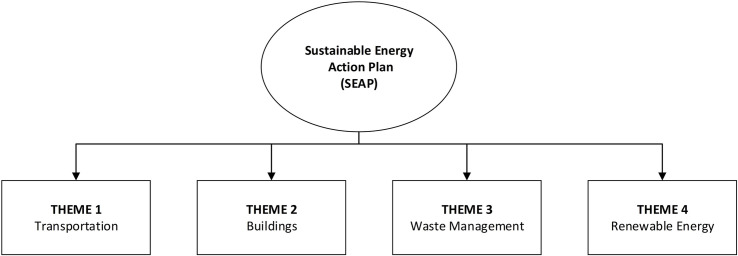
Themes of the Sustainable Energy Action Plan (SEAP).

The SEAP has four main themes (transportation, buildings, waste management, and renewable energy), as demonstrated in Figure 2.

The components of the SEAP are as follows: zero-emission public transportation project, transformation of existing buildings to a more energy-efficient standard and related energy audit studies, a project for reducing traffic wait time *via* a Smart Traffic System, a pedestrianization project, and a project for promoting geothermal energy for district heating.

In what follows, we track the footprints of the SEAP in order to identify the internal and external dynamics in operation during the process, *via* expert interviews designed to elicit different viewpoints and perceptions of stakeholders from different levels of the municipality. The scope starts with the decision to sign up to the Covenant of Mayors and is slightly extended to future projections regarding the SEAP and its sub-projects in order to identify the mechanisms, motivators, and barriers that influence the decision-making process under different circumstances and decision-making situations. An example of the different factors influencing these processes is the decision to become a signatory of the Covenant of Mayors. At first, this appeared to be a wholly top-management endeavor. It later emerged that mid-management was the initial origin, and the process was characterized by a bottom-up approach.

### Factors Affecting the Decision of Undertaking the Endeavor

#### Internal Dynamics

The Covenant of Mayors is the most wide-ranging framework, including large-scale governments, and local authorities at the national scale; this was a factor in the decision to become a signatory. It was also considered a means of increasing the domain of command for local governments and promoting improvement in their decision-making processes.

*“We became a party to the Covenant of Mayors since it was very common, and it had a lot of members. Moreover, it had a specific framework and constituted a better guideline for us [*…*] Many colleagues said that the Covenant of Mayors would ensure privilege and priority to take grant for EU projects.”*

*Interview I1*

*“Consequently, the local authorities have more quick and efficient decision-making processes regarding environmental issues. Therefore, there is a bottom-up decision-making structure. The local authorities do not wait for any instruction from the top management. If every local authority fulfils its duty, the world will be able to find a proper solution for these environmental problems.”*

***Interview I2***

*“In 2015, we attended the Paris Climate Conference. In the conference, it was revealed that the global problem caused by climate change could only be solved with the projects conducted by local authorities [*…*] As we believed that municipalities were highly effective in creating a climate-resistant city, we decided to launch the projects for the fight with climate change.”*

***Interview I1***

Although local governments have experienced the benefits of a leaner organizational structure that encourages more active and well-organized decision-making processes, its hierarchical structure can occasionally slow the decision-making process. This structure includes bottom-up and top-down mechanisms; for example, decisions must have the approval of the Municipal Council, as an authority above the Mayor.

*“In 2015, the Municipality became a party to the Covenant. The decision was presented to the Municipal Council; the Mayor signed the document and approved it. The Municipal Council is above the authority of the Mayor [*…*] Similarly, the Municipal Council in the city is accepted as the representative of all the citizens living in this city. Therefore, the decision of the Council constitutes a great significance. Only the Mayor’s decision is not sufficient to become a party to these kinds of agreements.”*

***Interview I2***

***“****Our commitment of a 20% emission reduction target was firstly approved by the Municipal Council. Then, the Council was convened and presented this issue to the Subcommittee of Environment. Currently, we have a policy document approved by the Municipal Council. This document became an official document approved by the top management. Therefore, our decision-making mechanism had a bottom-up structure, but the top management became conscious to a great extent.”*

***Interview I4***

In general, the interviewees agreed on the vital role of both top-down and bottom-up mechanisms. This emphasizes the important influence of the internalization of the residents’ voice, and also the mid-level managers’ efforts. The top management had two roles: taking the final decision and fostering the implementation.

*“[*…*] our Mayor became a pioneer for the other 11 municipalities regarding the implementation of the Sustainable Energy Action Plan. Our Sustainable Energy Action Plan was a precondition to participate in other European Union H2020 projects. These preconditions include the design of Action Plans, emission reduction targets, an inventory study to achieve these targets, declaration of intention and a comprehensive report regarding the actions to be taken.”*

***Interview I3***

*“It was a project that came from the bottom of the structure of the firm. When the general manager accepted this, the Covenant of Mayors supported this project [*…*]. The project idea came from the bottom, but it was a good thing that we had an assistant general manager supporting us.”*

***Interview I5***

#### External Factors

##### The role of central government

The perception of the overwhelming authority of the central government emerged as a crucial factor. In Turkey’s management culture, the decision-making dynamics is usually considered to have a top-down structure, underlining the role of central government as a powerful actor both in decision making and in planning and implementation.

*“As there is a strong central government in our country, the decision-making mechanism has a top-down structure. This means the plans and projects are designed by top level executives. Therefore, we can achieve all plans with the support of external stakeholders such as the Ministry of Energy and the Ministry of Environment and Urbanization.”*

***Interview I3***

*“The Covenant of Mayors offers an option for us regarding the decrease of carbon emission. Accordingly, there are sectors that are under the responsibility of Municipalities. However, the industrial sector is not under the responsibility of Municipalities.”*

***Interview I2***

##### Perception of side benefits

Municipality authorities expected membership in the Covenant of Mayors to have lateral benefits, for instance, supporting municipality’s applications to further EU project grants.

*“Furthermore, we can get EU projects by way of the Covenant of Mayors. Many colleagues said that the Covenant of Mayors would ensure privilege and priority to take grant for EU projects.”*

***Interview I1***

### Factors Affecting the Planning and Implementation Phases

The methodology for these Action Plans was initially established by the Covenant of Mayors itself. Succeeding the course of action as recommended by the Covenant of Mayors seemed candid.

*“Our Mayor became a party to the Covenant of Mayors and gave a commitment to reduce carbon emission by 20% until 2020. As a part of this commitment, we had to prepare a SEAP. Now, the Action Plan is in the implementation process.”*

***Interview I1***

*“After we became a party to the Covenant of Mayors, we gave a commitment to prepare the Sustainable Energy Action Plan within 1 year. Therefore, we made an attempt for the preparation of the Plan. There is a methodology suggested by Covenant of Mayors for the preparation of this Plan. The Plan was designed according to this methodology.”*

***Interview I2***

However, various factors had noteworthy impacts on how these phases eventually evolved.

#### Internal Dynamics

The organizational construction and organizational capacity were found to be key to the planning and implementation stages. In this specific case, the organizational structure was modified by the creation of a department responsible for the SEAP, followed by the creation of an auxiliary project team from members of other divisions. Workshops were organized to build and improve organizational capacity.

*“We are the first municipality that established a Directorate on this issue. As I said before, we determined the carbon emission values of the city. Then, we determined what we could do within the scope of the municipality’s own responsibility for the reduction of carbon emission values, and we started to take actions.”*

***Interview I1***

*“All the departments in the municipality need a guidance for the implementation of these Action Plans. Therefore, we organized meetings to inform the employees in the municipality. We explained to them what the Sustainable Energy Action Plan is and why we conduct such a project.”*

***Interview I3***

*“The first thing to do was to encourage the stakeholders to adopt this plan. After the Sustainable Energy Action Plan was approved in the Municipal Council, we gathered only the top executives of all our companies and all our units in the assembly hall. The Mayor also mentioned the studies on the Sustainable Energy Action Plan and set some targets. In this way, all stakeholders started to focus on topics such as renewable energy and transportation.”*

***Interview I6***

The creation and development of bottom-up mechanisms by top-level managers were among the most substantial internal factors concerning implementation.

*“The key factor for the effectiveness of the Sustainable Energy Action Plan is the fact that decision-makers and top executives encourage and support the personnel. The decision-makers support and give importance to the ideas and suggestions put forward by the personnel in the bottom.”*

***Interview I2***

In as much as the SEAP is an effort linked to the municipality as a collective decision-making entity, the involvement of individuals and especially, of the Mayor, is vital in the introduction of this Action Plan.

*“The fact that Mayor was aware of the issue and supported us in the fight with climate change was the major effective factor. The city was a pioneer with respect to environmental issues and got a reward from the United Nations as the first environment friendly city*… *I think the key factor behind this success was based on high awareness level of the executives, their support and a competent technical team.”*

***Interview I1***

*“The Mayor made a speech on this issue and invited everyone to take charge of the implementation of this action plan. He also had an aim to encourage the municipality to generate its own electricity from renewable energy resources. In other words, the success of the project is based on the initiatives of the Mayor and commitment of the personnel.”*

***Interview I1***

The employees’ technical competence and self-assurance are likewise viewed as a significant factor in the implementation of the SEAP.

*“We work with employees that are technically self-conscious. They really have a high level of awareness; they like research and development; they want to improve themselves.”*

***Interview I1***

*“I think it is very useful in terms of raising awareness and developing a corporate perspective*… *I think SEAP is highly influential in terms of raising awareness of the technical personnel in the municipality. I believe that it will be quite successful in the end.”*

***Interview I1***

#### External Factors

The planning and implementation phases involved challenges over which the authority of the central government was decisive. A number of sectors evidently within the scope of the SEAP were excluded from the planning and implementation phases, which were under jurisdiction of the central government. This encompassed the industrial sector, which produced the largest share of carbon emissions at 50% of the total.

*“[*…*] We also set a target regarding renewable energy. At this point, there are two different sets of categories consisting of targets. One is the targets within the Municipality, and the other one is the targets on the urban scale. The targets on the urban scale are more flexible, and the industrial and agricultural sectors were excluded as the municipalities could not intervene in these sectors. However, the inventory study included these two sectors.”*

***Interview I4***

The participation of external stakeholders is critical for the implementation of the SEAP. To this end, a workshop was organized for the participation of external stakeholders and capacity building.

*“A workshop was organized in the most critical stage of this project. All the related stakeholders including universities, trade associations, the Department of Transportation, the Chamber of Civil Engineers, the Chamber of Survey Engineers, and the Chamber of Architects were invited to the workshop. The most important aspect of organizing such a workshop was to see the projects of the external stakeholders.”*

***Interview I3***

Public awareness and contribution are critical for the implementation phase. To achieve carbon emission reduction targets, sections of the SEAP include policies directly targeting citizens.

*“[*…*] we designed projects to achieve [*…*] emission reduction targets. The projects included studies to raise awareness about solar energy projects, waste management, transportation, pedestrianization and use of bicycles, electric vehicles and electric buses.”*

***Interview I3***

*“One aim of transportation planning is to popularize the public transportation options such as the metro and tram, as well as pedestrian sidewalks and bicycle roads.”*

***Interview I6***

**FIGURE 3:**
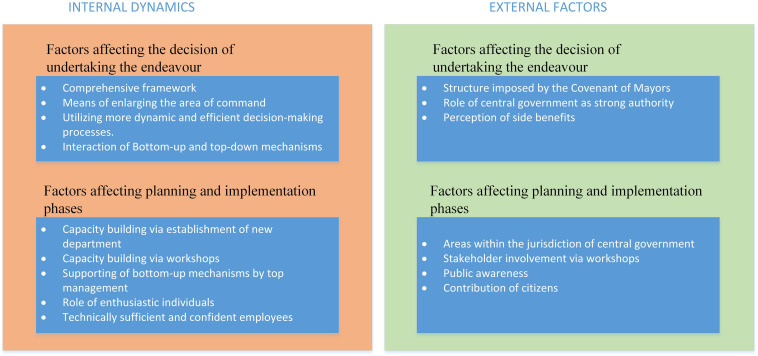
Internal dynamics and external factors affecting the phases of Sustainable Energy Action Plan (SEAP).

Figure 3 provides a summary of the internal dynamics and external factors affecting the decision to undertake the responsibility for planning and implementation of the SEAP in its various phases.

### Evidence From Implementation, Barriers, and Motivators

#### Results of the Implementation

Considering the outcomes of the implementation of the SEAP, the key result is the achievement of energy savings and environmental targets.

*“Energy efficiency does not only provide a financial opportunity and saving but also provides a chance to reduce carbon emissions. Moreover, we decrease our energy consumption in this way.”*

***Interview I4***

*“It is possible to make energy saving by 5% only by informing people and raising their awareness.”*

***Interview I6***

*“In 2019, electric vehicles will be operating in 4 different cities. Interestingly, all the municipalities in these cities purchased 20 electric vehicles. I think they take this city as an example. We believe these projects will increase in local governments.”*

***Interview I5***

Stakeholders’ increasing awareness and support, and enthusiasm for further actions also boost energy-saving behavior.

*“Both the management and public are more aware of these issues. As the public awareness has increased regarding climate change-related issues, the popularity of Sustainable Energy Action Plan has started to rise. These kinds of Action Plans constitute an umbrella for all other plans, programs and strategies of the municipalities.”*

***Interview I4***

*“As our effort is adopted by the top management, all stakeholders are more enthusiastic about developing these kinds of projects.”*

***Interview I3***

*“Now, we can see that all other departments try to take a step in this field without our intervention. This implies that every department in the municipality has started to give more importance to energy-related issues and energy systems.”*

***Interview I1***

Related impacts on a greater scale are anticipated. These projects are expected to increase the awareness of the notion of sustainability nationally.

*“I think that these plans will further increase the importance of the concept of sustainability in multiple fields: sustainable energy activities, sustainable growth, sustainable development and sustainable cities.”*

***Interview I6***

*“We achieved our target to create a sustainable city by minimizing the effects of environmental pollution without depleting the natural resources. I am also very hopeful in the national sense and I think that these efforts will not be in vain.”*

***Interview I1***

Throughout the project, design, operation, and organizational capabilities were enhanced. This heightened know-how will be available in comparable upcoming projects, and the process was also important in revealing issues requiring further enhancement. Furthermore, the encouraging outcomes of the implementation can also be observed in external stakeholders, particularly suppliers that serve as the industrial partners.

*“If you make a good example of an implementation, you can definitely apply it to the others. Other municipalities realized this implementation here [*…*] Our project was the major experience for us.”*

***Interview I6***

Thus, the endeavor also provides avenues for further collaboration between public and private sectors for mutual benefits of the stakeholders.

*“We will probably have to focus more on battery management system*… *The infrastructure of these battery management systems will be firstly used applied in electric cars. Then, the buses and trucks will be supplied with the same system. Turkey is regarded as the commercial vehicle centre. We export many products to Europe from Turkey. This shows that our infrastructure is actually quite developed.”*

***Interview I6***

Another important impact is greater public acceptance.

*“We don’t have any hesitation regarding electric vehicles since we realized that they were quite economical. Our citizens use them and they have an experience regarding these vehicles. The only problem is the fact that they have started to normalize them. There is no obvious demand from the public regarding the increase of electric vehicles in public transportation.”*

***Interview I5***

**FIGURE 4:**
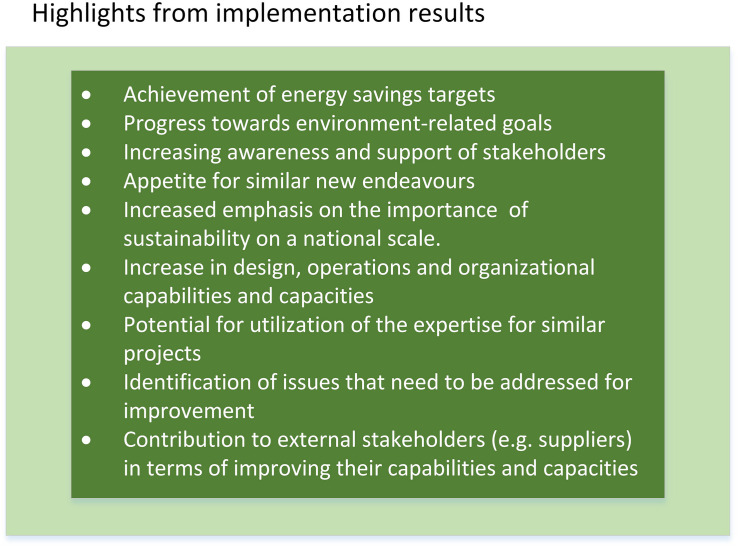
Highlights from implementation results of the Sustainable Energy Action Plan (SEAP).

Figure 4 reveals the key highlights from implementation outcomes of the SEAP.

#### Barriers

The interviews pointed out a number of barriers that delay or adversely affect the general process of the SEAP.

##### Lack of awareness and acceptance

Citizens are not familiar with the notions and issues associated with climate change, thus hindering the Action Plan adaptation process. As one solution, the Operational Employee proposes publicity campaigns to raise citizens’ awareness.

*“At the beginning, they had difficulty in conceptualizing carbon emission and the setting of emission reduction targets; however, they adopted this plan well after the public and non-governmental organizations became more aware of climate change-related issues.”*

***Interview I3***

*“We also advertised our emission reduction targets on the television news.”*

***Interview I3***

##### Resistance to change and slow social progress

Resistance to change is observed as additional key barrier to implementation, as it obstructs the social progress toward a low carbon city. NGO publicity campaigns and activities will be significant in reducing this resistance.

*“As a matter of fact, some of the people show resistance to the measures taken against climate change while some other accept the struggle with climate change as a facilitating trend. The civil society organizations are also quite successful in this regard. They organize campaigns and direct people to take an action against climate change.”*

***Interview I4***

*“I don’t feel very hopeless, but I see that our social progress is unfortunately very slow*… *We lost the enthusiasm of the citizens because of the problems caused by the citizens and the handicaps in the state administration.”*

***Interview I1***

##### Bureaucratic issues

Three of the interviewees identified three main types of bureaucratic barriers: regulatory issues, administrative boundary problems, and the role of central administration.

The main difficulties concerning regulations in Turkey are their frequent amendments, the multifaceted legal structure, and long-established legal procedures, in addition to shifting political conditions. These factors present challenges in the implementation of the Action Plans.

*“The regulations restricted us regarding the implementations in renewable energy systems. They made their implementation difficult at the national level.”*

***Interview I2***

*“As far as the legislation is concerned, it is seen that the legislation was the common problem, because it is constantly changing, and it has a complex structure.”*

***Interview I3***

*“We improved our system in terms of regulations, because the regulations in Turkey are severe on this issue and the legal procedures take a long time.”*

***Interview I3***

The comprehensive scope of the applied regulations denies any opportunity for privileges or exemptions for different types of organizations.

*“For example, we had a correspondence with General Directorate of Renewable Energy on this subject, but we did not get a positive result. We said that the regulations make it impossible to conduct such kinds of projects. We also asked whether there is any privilege for the municipalities and official institutions. As a municipality, we said that our buildings had a different position and offsetting was very economical for us. However, they said there was no privilege on this issue.”*

***Interview I1***

An additional regulatory problem concerns the administrative boundary; i.e., the provincial boundaries of the city were enlarged by central government regulations. These affected the size of the population and extended the control area, creating challenges for the Metropolitan Municipality in achieving targets.

*“The provincial borders of the city were changed as provincial administrative boundaries. Consequently, the responsibility of the district municipalities was transferred to the Metropolitan Municipality and the consumption level of the Metropolitan Municipality increased.”*

***Interview I4***

*“We’re having a hard time in explaining these facts to the citizens, because the population is quite high in large cities.”*

***Interview I6***

Another challenge is the crucial role of central government, and particularly the Municipality’s economic dependence on the central administration.

*“There is an engagement in the sense of economic commitment to central administrations and compliance with the decisions made. This might be the major challenge in practice.”*

***Interview I1***

Despite the existence of incentive mechanisms in Turkey, a number of institutions have difficulty in making investments in renewable energy systems. Local authorities cannot fully benefit from these mechanisms due to the complexity of the procedures.

*“We were very disappointed, because while establishing the solar power plants, the central administration receives certain costs related to the transformer connections. These prices have been increased by 13 times. Therefore, installation of rooftop solar panel and offsetting of a different building was not a profitable and economical decision anymore. At this point, I lost my interest and enthusiasm to install solar panels on our buildings.”*

***Interview I1***

##### Issues with data collection

As stated in the roadmap, a full inventory study and precise data collection are important for effective implementation. Yet a noteworthy barrier remains in the form of challenges in the data-gathering process, together with the data’s technical insufficiency and inarticulateness, in terms of both national and international standards.

*“In the urban scale, local electricity distribution companies became a stakeholder in the project. In this regard, the Mayor made several correspondences with these stakeholders. In fact, we had difficulty in collecting data from external sources [*…*] This is probably caused by the fact that electricity distribution companies have been privatized and this data can be collected from private companies. These challenges might stem from resistance, unwillingness, or commercial factors.”*

***Interview I6***

*“Within the framework of the Sustainable Energy Action Plan, they asked us how many universities and personnel work for the fight with climate change in the city. We don’t have any data on this issue.”*

***Interview I3***

*“Another challenge was the fact that the partners and stakeholders in the project did not have technical adequacy and they could not keep data regularly.”*

***Interview I4***

##### Financial issues

Costs and budget considerations cause great difficulty in the implementation of renewable energy developments. Currently, there are inadequate incentive mechanisms to boost domestic investment in renewable energy systems in Turkey.

*“The cost of generating energy from renewable energy resources is pretty high. We have one more problem: Who will cover the expenses of such a renewable energy project?”*

***Interview I3***

*“As the return on investment period is too long and costs are extremely high, some of these systems are no longer profitable and economical. Although some of our existing roof areas were available, we had to reduce the number of solar panels and started to use them only for meeting the consumption of the building.”*

***Interview I1***

*“The price of one electric bus equals to the price of three conventional fossil fuel-based busses. At this point, the question is that whether we should provide a cleaner service to less people, or an environmental polluting service to more people.”*

***Interview I2***

One further problem is associated with the difficulty in accessing sufficient financial resources. In the case of a developing country, exchange rate volatility and the lack of technological adaptation pose a vital challenge for sustainable energy projects at both local and national levels.

*“Especially Europeans question many things regarding their financial sources, and they have sufficient financial sources for renewable and sustainable energy projects. The financial issues are also extremely significant for us. Besides financial sources, they have the technology to successfully conduct these kinds of projects [*…*] In fact, increasing foreign exchange rate creates serious problems for us in terms of financing these kinds of projects. Turkey is a developing country and we need financial support and efficient financial sources.”*

***Interview I3***

*“It was suggested that we could install solar panels on the rooftop in the urban scale, but we realized that these kinds of large-scale projects would require huge investments. Therefore, they were not feasible.”*

***Interview I6***

#### Motivators

As well as facing barriers, the SEAP process has also benefited from motivating factors regarding the implementation. The following section analyzes the major positive as identified in the interviews.

##### Advantages of being a local authority

In addition to the lean organizational structure and dynamic decision-making process, the municipality has advantages of a local authority’s close relations with the public, and its ability to foster cooperation with the Mayor and other municipal stakeholders.

*“As a local authority, we have many advantages. We can create close relations with the public. As we train our employees in the municipality, they can also convey their own experiences to the public.”*

***Interview I4***

*“The most important thing that makes our work easier is the importance given by our top management in this city to climate change-related issues.”*

***Interview I3***

##### The perceived goal of living in a cleaner city

The implemented Action Plan allows a greener, cleaner, and more sustainable environment, providing citizens with prospects for healthier environmental conditions.

*“These projects may enable a cleaner and livable city. This is the major motivator for all citizens.”*

***Interview I6***

*“The citizens in the city are more sensitive. This is the most important point that differentiates the citizens of this city from the others*… *They say that it is much easier to spread an implementation to all other cities if it is firstly applied in this city.”*

***Interview I3***

##### Affiliation with international organizations

As climate change is a global problem that affects all humans worldwide, it is of great importance to work within international organizations and simultaneously to consider national interests. These memberships stem from the motivation for joint action on climate change.

*“We are one of the members of World Healthy Cities Association. We believe that our membership is an encouraging attempt.”*

***Interview I1***

*“We decided that the Covenant of Mayors would be a good motivating factor for us in this process. We put forward a suggestion on this issue to our executives. The Municipality had already a positive attitude toward these kinds of initiatives.”*

***Interview I2***

##### Top-management vision and commitment

*“Our executives have always highlighted that in the next 10 years, the electric vehicles will be on our agenda; especially in the next 20 years they will be seriously prioritized. We should make our research about this issue well; we should keep our data properly; as these things develop, there will be serious changes in our energy infrastructure. It was explained that we had to create units and departments that would follow this process. It is even planned to create other units such as a Directorate of Energy and a Department of Energy.”*

***Interview I5***

##### Teamwork and enthusiastic personnel

*“Our assistant general manager was leading the project. There is an ongoing electric vehicle project in our company, and also a commission was established including young engineers dealing with the supply of electrical equipment from different branches.”*

***Interview I5***

###### Bottom-up mechanisms

*“It was a project that came from the bottom of the structure of the firm. When the general manager accepted this, Covenant of Mayors supported this project. Then, it was asked why the project was not combined with solar energy plant project. The project idea came from the bottom, but it was a good thing that we had an assistant general manager supporting us.”*

***Interview I5***

**FIGURE 5:**
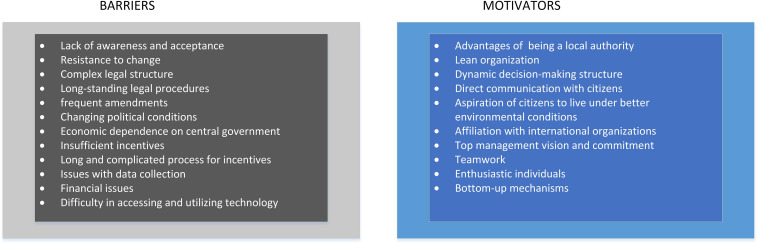
Barriers and motivators of the Sustainable Energy Action Plan (SEAP).

Figure 5 provides a listing of barriers and motivators of the process of SEAP.

## Conclusion, Discussion, and Recommendations

Izmir Metropolitan Municipality’s environmental protection and sustainability initiatives provide an exemplary case that can guide other local administrations and collective decision-making units. The process is conducted in significant phases, each involving different aspects, from the initial idea to implementation, through post-implementation results. Although often perceived as a straightforward implementation of the Covenant of Mayors, in fact, each step demonstrates a complex interaction between mechanisms, influencing factors, barriers, and motivators. These parameters extend across a wide spectrum of themes, including infrastructural, organizational, social, administrative, individual, and financial aspects. The identification of these characteristics allows for the evaluation of the process, and this allows learning that can be applied in similar implementations. It also points out the multidimensional nature of such a process and highlights the need for a comprehensive approach.

Expert interviews and case study methodology were implemented for the analysis and identification of relevant factors. The involvement of interviewees from different backgrounds and managerial levels enhances representativeness and ensures the incorporation of a range of stakeholder viewpoints and perceptions.

As a result of the outputs of the interviews, several suggestions and recommendations can be identified for the success of the SEAP and similar endeavors.

An overview of the SEAP process marks the vital role of top-level management. In particular, the commitment of top-level management is crucial in the initial phases of the process and for ensuring organizational acceptance. In some cases, the top-management commitment is driven by environmental factors but in others by personal or corporate reputation, which can be perceived as a driver pertaining to Turkish culture. The top-level management also significantly contributed toward establishing the balance between the framework drawn by the central government and its application by local authority. In parallel with the evidence from the literature, the SEAP experience emphasizes that the central government’s considerable jurisdiction is the determining factor in terms of legal and administrative restrictions and budget. Other outcomes of the government’s prevalent power are the underutilization of the potential synergy between the local and central governments, and the inability of local governments to develop and implement policies customized for the requirements of their communities. Such challenges are alleviated to some extent by memberships to initiatives such as the Covenant of Mayors.

The case also verifies findings from the literature revealing that such initiatives cannot be successful without encouraging and operationalizing bottom-up approaches. There are numerous factors affecting citizens’ acceptance of energy initiatives and their contributions to these initiatives. The literature includes numerous studies emphasizing the importance of social and cultural aspects, such lifestyle choices and habits, for such initiatives. Emphasis on the economic benefits for the city and individuals also contribute to increasing awareness and building trust. In the case of the SEAP, public awareness and contribution have only been addressed during the implementation phase; however, this study points to the need for policies and measures to increase citizens’ awareness, trust, and contributions from the design phase.

The technical and administrative capacities of the organization also play a key role as a success factor. Qualified, competent professionals and experts are needed to further contribute to environmental initiatives.

Another component pertains to collaboration with external stakeholders such as industry and research institutions. A significant opportunity is provided by public–private partnership mechanisms, which can be effectively encouraged by cases similar to the zero-emission transportation component of the SEAP. In such cases, municipalities or governmental institutions should seek collaboration with local industries or startups through the development of new products, services, or technologies, such as battery management software for electric public transport vehicles. In return, the community receives custom solutions that fit their specific requirements. Public–private partnerships can be developed by not only local-scale initiatives but also subsidies and research and development (R&D) funding schemes. Additionally, trilateral cooperation between the university, industry, and public should be encouraged *via* mechanisms such as workshops or meetings. This local collaboration, often overlooked in the existing literature, also promotes investment in R&D for technological improvement and domestic production.

Another finding from the analysis of the SEAP case is that countries that lag behind contemporary technological advances will inevitably depend on external sources, material, and expertise. This hinders the successful implementation of decisions due to greater costs and a narrower set of potential alternatives.

Existing studies in the literature suggest that, under the current paradigm, governments and local authorities focus on climate change mitigation rather than adaptation activities. However, there is a need for improvement in adaptation, based on future projections in terms of how countries, as well as cities and urban places, might react to these negative impacts. Accordingly, municipalities and local governments need to incorporate the regional climate model projections into their adaptation strategy and preparations. The SEAP also provides an example in which motivators, such as the Covenant of Mayors, encourage local governments to set goals more ambitious than those of central governments and then formulate and implement initiatives to achieve these.

The SEAP and similar plans involve action plans and projects. The success of the SEAP is highly dependent on how far these plans and projects are designed for and tailored to the needs of the local community. Equally important is the inclusion of well-defined follow-up and monitoring mechanisms to track the progress of implementation. The feedback process embedded in these mechanisms allows identification of issues and setbacks with implementation and facilitates the deployment of appropriate actions.

The implementation and success of initiatives such as the SEAP rely on several key factors, as emphasized in the interviews. To begin with, undertaking such an endeavor is more challenging without similar earlier implementations because supporting stakeholders, e.g., side industries, are not familiar with the requirements or specifications expected of them. Therefore, emphasis is placed on networking, experience sharing, learning from best practices, and even from failures. *A priori* information on potential barriers may help cities and also industries in designing remedies, just as an understanding of the toolbox of motivators may provide opportunities for better exploitation.

## Data Availability Statement

The data generated for this study are available on request to the corresponding author.

## Ethics Statement

The studies involving human participants were reviewed and approved by İzmir University of Economics’ Ethics Committee for Social Sciences. The patients/participants provided their written informed consent to participate in this study.

## Author Contributions

MEB and MHD conceptualized the research methodology, interpreted the results together, and wrote the “Conclusion, Discussion, and Recommendations” section. MEB designed the manuscript, undertook fieldwork *via* contacting six municipality representatives, and conducted the interviews together with MHD. MHD performed the analysis and prepared the first draft. MEB revised the first draft, updating and writing the developing sections of the manuscript. Both authors have read and approved the final manuscript.

## Conflict of Interest

The authors declare that the research was conducted in the absence of any commercial or financial relationships that could be construed as a potential conflict of interest.
